# Serum Carotenoids Are Inversely Associated with RBP4 and Other Inflammatory Markers in Middle-Aged and Elderly Adults

**DOI:** 10.3390/nu10030260

**Published:** 2018-02-25

**Authors:** Lipeng Jing, Mianli Xiao, Hongli Dong, Jiesheng Lin, Gengdong Chen, Wenhua Ling, Yuming Chen

**Affiliations:** 1 Department of Medical Statistics & Epidemiology, School of Public Health, Sun Yat-sen University, Guangzhou 510080, China; jinglp@mail2.sysu.edu.cn (L.J.); xiaomli@mail2.sysu.edu.cn (M.X.); dhljiyi@163.com (H.D.); linjsh6@mail2.sysu.edu.cn (J.L.); chgengd@163.com (G.C.); 2 Guangdong Provincial Key Laboratory of Food, Nutrition and Health, School of Public Health, Sun Yat-sen University, Guangzhou 510080, China; lingwh@mail.sysu.edu.cn

**Keywords:** carotenoids, inflammatory marker, antioxidant, retinol binding protein 4

## Abstract

(1) Background: Carotenoids may be inversely associated with inflammatory markers (i.e., TNF-α, IL-6, IL-1β). However, data are scarce on retinol binding protein 4 (RBP4) in humans. We examined the associations among serum carotenoids, RBP4 and several inflammatory markers in a Chinese population. (2) Methods: This community-based cross-sectional study included 3031 participants (68% males) aged 40–75 years in Guangzhou, China. Serum concentrations of carotenoids, RBP4, and inflammatory markers were measured. (3) Results: Generally, serum individual and total carotenoids were significantly and inversely associated with retinol-adjusted RBP4, RBP4, hsCRP, MCP1, and TNF-alpha levels. Age- and gender-adjusted partial correlation coefficients between total carotenoids and the above inflammatory markers were −0.129, −0.097, −0.159, −0.079, and −0.014 (all *p* < 0.01, except for TNF-alpha with *p* >0.05), respectively. The multivariate-adjusted values of partial correlation coefficients for these inflammation-related markers were −0.098, −0.079, −0.114, −0.090, and −0.079 (all *p* < 0.01), respectively. Among the individual carotenoids, those with the most predominant association were lutein-zeaxanthin and total carotenoids for retinol-adjusted RBP4 and RBP4, alpha- and beta-carotene for hsCRP, and alpha-carotene for MCP1 and TNF-alpha. No significant associations were observed for IL-6 and IL-1beta. (4) Conclusions: Serum carotenoids were inversely associated with RBP4, hsCRP, MCP1 and TNF-alpha among middle-aged and elderly Chinese adults.

## 1. Introduction

Inflammation is an essential component of immunosurveillance and host defense. However, a chronic increase in inflammatory markers, such as interleukin (IL)-6, IL-1, tumor necrosis factor α (TNF-α) [1], and C-reactive protein (CRP) [2,3], plays a key role in various chronic conditions (e.g., type 2 diabetes mellitus [T2DM], cardiovascular diseases [CVDs] [4,5], and cancer [6]). Controlling systematic inflammation would thus be a targetable approach in the prevention of these chronic diseases.

Fruits and vegetables are rich in carotenoids, a group of fat-soluble phytochemicals. The levels of blood carotenoids are inversely associated with risks of many chronic diseases, including T2DM [7,8,9], CVDs [10,11,12], and several types of cancers [13], and carotenoids act by reducing systemic inflammation [14]. Animal studies have shown a favorable effect of carotenoids on inflammatory markers (i.e., TNF-α, MCP1, VCAM-1, IL-6, IL-1β) [14], in which much higher doses than for normal human consumption were used. This favorable association was also detected in several cross-sectional studies [15,16,17] and prospective studies [18,19]. However, these studies mainly focused on CRP, TNF-α, and IL-6. Given that different inflammatory markers may play independent roles and affect different pathways in the development of chronic diseases [20], further studies are needed to assess the association between serum carotenoids and many other inflammatory markers (i.e., MCP1, IL-1β) in humans. 

Retinol binding protein 4 (RBP4) is the sole retinol transporter in the blood and is secreted from the liver and adipocytes [21] (also known as a novel adipose-derived cytokines). RBP4 was speculated to be an important proinflammatory marker by inducing inflammation by activating JNK, p38 MAPK signaling, and NF-κB pathways [22,23,24], as well as increasing secretion of TNF-α, IL-6, and IL-1β. In addition to its proinflammatory function, RBP4 mainly causes insulin resistance [25] and is linked to various chronic diseases [26,27,28]. A few animal studies showed that antioxidants (e.g., resveratrol [29], cyanidin 3-glucoside [30], green tea polyphenols [31]) down-regulated RBP4 expression in white adipose tissue [29,30] and inhibited the overproduction of plasma RBP4 [31]. In light of the antioxidative feature of carotenoids, it is reasonable to hypothesize that elevated serum carotenoids may be beneficial in decreasing the levels of circulating RBP4. However, the relationship between carotenoids and RBP4 has been explored only in a secondary analysis of a trial supplemented with fruit and vegetable juice concentrate in 30 prepubertal boys. In this study, a null association was observed between the blood levels of β-carotene and RBP4 [32]. To our knowledge, no study has yet assessed this association in any adult population.

To address the above questions, this community-based cross-sectional study mainly examined the associations of five major serum carotenoids (α-carotene, β-carotene, β-cryptoxanthin, lycopene and lutein/zeaxanthin, as well as total levels) with RBP4 and hsCRP, a traditional inflammatory marker, and examined the associations with a variety of other inflammatory markers including MCP1, IL-1, IL-6, and TNF-α, in a middle-aged and older Chinese population.

## 2. Materials and Methods

### 2.1. Participants

Participants included in this community-based cross-sectional study were from The Guangzhou Nutrition and Health Study (GNHS); the study enrolled 3169 participants from 2008 to 2010, and 832 new subjects were recruited in 2013 in Guangzhou, China. Participants with cancer, CVD, stroke and mental illnesses were excluded. Furthermore, participants with missing carotenoid and inflammatory markers were excluded. Finally, 2925 participants with serum carotenoid data and RBP4 or at least one inflammatory marker were included in this study. Written informed consent was obtained from all of the subjects. This study has been registered at www.clinicaltrials.gov as NCT03179657. The study protocol was approved by the Ethics Committee at Sun Yat-sen University.

### 2.2. Data Collection

The following information from the participants was obtained: socio-demographic characteristics, lifestyle factors, smoking and drinking habits, and multivitamin and calcium supplementation; the data were collected using a face-to-face interview with a structured questionnaire. The metabolic equivalent (MET) was calculated from a 19-item physical activity questionnaire [33]. Height and weight were measured, and body mass index (BMI) was then calculated as weight/height^2^ (in kg/m^2^). The waist circumference (WC) was also measured.

### 2.3. Measurement of Serum Carotenoids

Venous blood samples were collected after an overnight fast. Serum was separated by centrifugation at 1500× *g* for 15 min at 4 °C, and the samples were stored at −80 °C until further analysis. Serum total cholesterol (TC) levels were measured by colorimetric methods using commercial kits (Biosino Biotechnology Company Ltd., Beijing, China) in a Hitachi 7600-010 automated analyzer (Hitachi, Ltd., Tokyo, Japan).

**Figure A1:**
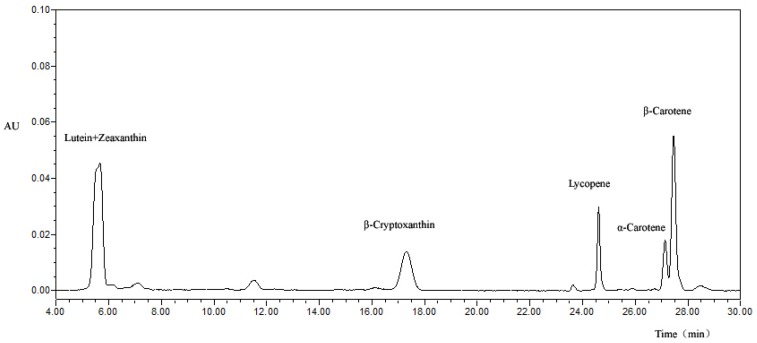
Chromatograms of serum carotenoids.

Reversed-phase high-performance liquid chromatography (HPLC) was used to simultaneously measure the serum levels of α-carotene, β-carotene, β-cryptoxanthin, lycopene and lutein/zeaxanthin with the methods described by Burri et al. [34] and with some modifications [35]. The mobile phase A contained acetonitrile/tetrahydrofuran/methanol/ammonium acetate (85:5:5:5, *v*/*v*), and phase B contained acetonitrile-methanol-tetrahydrofuran-ammonium acetate 55:35:5:5, *v*/*v*. The Waters 2998 diode-array detector was used (Waters, Milford, MA, USA). Then, the chromatograms of serum carotenoids was shown in Appendix Figure A1. The day-to-day coefficients of variation (CV) were 7.8% for α-carotene, 8.6% for β-carotene, 10.6% for lycopene, 9.7% for β-cryptoxanthin and 8.0% for lutein + zeaxanthin.

### 2.4. Measurement of RBP4 and Inflammatory Markers

Serum RBP4 levels were measured using the enzyme-linked immunosorbent assay (ELISA) kit (Adipogen, San Diego, CA, USA), and the absorbance value was determined by a microplate spectrophotometer (BIO-TEK, Winooski, VT, USA). The lowest level of RBP4 that can be detected by this assay is 380 pg/mL (between-plate CV: 3.59%). High sensitivity C-reactive protein (hsCRP) was measured using the Cardiac C-Reactive Protein (Latex) High Sensitive (CRPHS) kit in the cobas c701 autoanalyzer according to the instructions by the manufacturer (inter-assay CV: 2.07%).

Serum levels of monocyte chemoattractant protein-1 (MCP1), tumor necrosis factor (TNF-α), Interleukin-6 (IL-6), Interleukin-1β (IL-1β) were measured in approximately 1/3 of the total subjects selected from one of three batches of serum samples according to the batch number sequence. Human FlowCytomix (Simplex BMS8213FF and BMS8288FF, eBioscience, San Diego, CA, USA) and the Human Basic Kit FlowCytomix (BMS8420FF, eBioscience, San Diego, CA, USA) on a BD FACSCalibur instrument (BD Biosciences, Franklin Lakes, NJ, USA) were used for the measurements [36]. The between-plate CVs were 14.1% for MCP1, 6.6% for TNF-α, 2.5% for IL-6, and 10.2% for IL-1β.

### 2.5. Statistical Analysis

Continuous variables were presented as the means ± standard deviation for normally distributed data or the median and the 25th–75th percentile ranges for data with a skewed distribution. The *t*-test or Mann-Whitney *U* test was used to compare the characteristics of the participants. For the categorical variables, the data were presented as frequencies (percent) and were compared using Chi-square test.

Logarithmic transformed data of the skewed distribution variables (TG, carotenoids, MCP1, IL-6, IL-1β, TNF-α, and hsCRP) and their Z-scores were then used in further correlation analyses. Since serum RBP4 levels are positively correlated with retinol levels, in this study, we also used crude RBP4 and retinal-adjusted RBP4 (aRBP4) as was done in a previous study [37].

Partial correlation analyses were used to assess the associations of the concentrations of total or each individual carotenoid with RBP4, retinol-adjusted RBP4, and inflammatory markers. The first model was adjusted for age and gender. Multiple models were further adjusted for BMI, TC, marital status, education level, household income (Yuan/month per person), smoking and drinking habits, vitamin supplements, and physical activity in all subjects and in women and men separately.

Further, concerning the biological relevance, we used logistic regression model to determine the association between serum concentrations of carotenoids and elevated inflammation levels which determined by hsCRP (≥1.0 mg/L) [38] and RBP4 (arbitrarily defined as top quintile by sex). The same covariates to those in the partial correlation analyses were included in the logistic regression analyses. *p* < 0.05 was considered statistically significant. All of the analyses were conducted using SPSS16.0 software (SPSS, Inc., Chicago, IL, USA).

## 3. Results

The characteristics of the study participants are shown in Table 1. Compared to men, women were of younger age and had lower BMI, proportion of smokers and drinkers, and concentrations of IL-6, IL-1β, TNF-α, and RBP4. However, women had higher values of physical activity, proportion of calcium and multivitamin supplements users, serum concentrations of carotenoids, TC, MCP1, and hsCRP (all *p* values < 0.05).

The associations between serum values of carotenoids and RBP4 or inflammatory markers are shown in Table 2. In general, both the age- and gender-adjusted model (Model 1) and multiple variable-adjusted models (Model 2) showed inverse associations for both total and specific carotenoids with retinol-adjusted RBP4, RBP4, hsCRP, MCP1 and TNF-α. Mean partial correlation coefficients of five carotenoids and the coefficients for the combined carotenoids (total) were −0.103, −0.077, −0.122, −0.062 and −0.008, respectively, in Model 1, and −0.077, −0.061, −0.085, −0.068 and −0.058, respectively, in Model 2 for the above five inflammation-related markers. Among the inflammatory markers, retinol-adjusted RBP4 and hsCRP had the most pronounced inverse associations, followed by TNF-α and MCP1, but there was no significant association for IL-6, and IL-1β (except for α-carotene). The carotenoids with the most predominant associations were lutein-zeaxanthin and total carotenoids for retinol-adjusted RBP4 (*r* = −0.140 and −0.120) and RBP4 (*r* = −0.093 and −0.084), α-carotene and β-carotene for hsCRP (*r* = −0.161 and −0.112 for α-carotene; *r* = −0.161 and −0.105 for β-carotene), and α-carotene for MCP1 (*r* = −0.088 and −0.090) and TNF-α (*r* = −0.080 and −0.096) in Models 1 and 2 (all *p* < 0.005).

The associations among serum carotenoids, RBP4 and inflammatory markers by gender are shown in Table 3. In the multiple variable-adjusted model, the favorable associations tended to be more pronounced for aRBP4 and RBP4 in women than in men, and MCP1 and TNF-α in men than in women. The significant correlation among IL-1β, α-carotene (*r* = −0.091, *p* < 0.01) and lycopene (*r* = −0.069, *p* < 0.05) was observed in women. The correlation between carotenoids and hsCRP tended to be similar in women and men. Furthermore, we adjusted for medications (e.g., statins, anti-hypertensive or anti-diabetic drugs), which could affect the concentration of inflammatory markers; the associations that were examined were slightly attenuated (Table A1).

We further explored the associations between serum levels of carotenoids and elevated hsCRP and RBP4. Multivariate-adjusted ORs (95% CI) of elevated hsCRP and RBP4 for each unit (log μmol/L) increase in total carotenoids were 0.42 (0.29–0.61) for hsCRP and 0.43 (0.27–0.67) for RBP4. For individual carotenoids, the corresponding ORs ranged from 0.48 to 0.76 for hsCRP (all *p* < 0.05 except for lutein+zeaxanthin) and from 0.44 to 0.75 for RBP4 (all *p* < 0.05 except for β-Cryptoxanthin) (Table A2).

## 4. Discussion

In this community-based cross-sectional sample of 2925 middle-aged and older women and men, we first found that serum carotenoid concentrations were inversely associated with retinol-adjusted RBP4, RBP4, hsCRP, MCP1 and TNF-α in general adults.

### 4.1. Carotenoids, Other Dietary Factors and Blood RBP4

RBP4 plays a causal role in insulin resistance by reducing immunity and inflammatory mechanisms in adipose and vascular tissues [24,25,39]. RBP4 is also linked to many chronic diseases, such as T2DM, CVDs [26,27,28]. These studies provided a rationale for therapies and prevention of insulin resistance and inflammation-related chronic diseases aimed at lowering serum RBP4 levels. Dietary factors are important modifiable factors in the prevention and control of insulin resistance and inflammation. To date, however, limited studies have examined the associations of dietary factors with blood RBP4 levels in both animal and human studies. In vitro and animal studies have shown that some antioxidants (e.g., resveratrol [29], cyanidin 3-glucoside [30], green tea polyphenols [31]) down-regulated RBP4 expression in white adipose tissue [29,30] and inhibited the overproduction of plasma RBP4 [31]. In human studies, blood RPB4 levels were significantly decreased with lifestyle interventions including exercise programs and dietary control in children [40,41], hypocaloric low-fat diet in adults [42], Mediterranean diets in adults (37 ± 7 years) [43], and by vitamin D-fortified dough in type 2 diabetic subjects [44]. However, a 6-month supplementation with fruit and vegetable juice concentrates did not significantly affect RBP4 levels in 30 prepubertal boys; in addition, there was no significant association between the serum levels of β-carotene and RBP4 [32]. Although no study has reported the association between carotenoids and RBP4 in adults as of yet, our results were generally consistent with the findings of antioxidant interventions in animal studies. Further studies are needed to confirm our results due to the limited evidence available. 

The mechanisms underlying the favorable association between carotenoids and RBP4 remain unclear. An in vitro study showed that the antioxidant property of resveratrol decreases the cellular mRNA levels of RBP4 and the RBP4 concentration in the culture medium; resveratrol also reduces triacylglycerol content and the mRNA levels of lipogenic genes that are associated with the over-production of RBP4 [29]. The down-regulation of the RBP4 gene was also observed in the mesenteric white adipose tissues in diabetic mice treated with another antioxidant (anthocyanin) [30]. Carotenoids are a well-known type of antioxidant that is found at high levels in fruit and vegetables and RBP4 is a transport protein for retinol that carries retinol from the liver to the periphery in the form of retinyl esters and beta-carotene in chylomicrons. We proposed that the antioxidative property of the carotenoids might explain the observed favorable association. However, no study has directly addressed the underlying mechanisms of the favorable association between carotenoids and RBP4. Further biological studies are needed to clarify whether carotenoids may ameliorate blood RBP4 levels by down-regulation of the RBP4 gene. 

### 4.2. Carotenoids and Blood Inflammatory Markers

In this study, we detected the strongest inverse association between serum carotenoids and hsCRP among the inflammatory markers in all subjects and observed similar but relatively less pronounced associations with MCP1, TNF-α and IL-1β (but not IL-6) in a subset of samples. In line with our findings, the favorable associations between serum carotenoids and inflammatory markers were observed in several cross-sectional studies [15,17,45,46,47], in prospective studies in 1682 Scottish postmenopausal women (mean 54.8 years) [18], and in 4580 American young adults (18–30 years) [19]. However, intervention studies with carotenoid-rich foods or isolated carotenoids show conflicting results [14]; these findings may be due to the prooxidant or proinflammatory role of carotenoids at significantly high doses [48] or the insufficient synergistic effects with other nutrients normally present in whole food [49,50]. Our results supported the inverse relationship between carotenoids and inflammatory markers.

As summarized by Kaulmann and Bohn [14], carotenoids and their derivatives can down-regulate the transcription of inflammatory mediators (e.g., IL-6, IL-8, cyclooxygenase 2, inducible nitric oxide synthase (iNOS)) by inactivating the nuclear factor (NF-κB) and mitogen-activated protein kinase (MAPK) pathways that may be activated by specific (TNF-α, IL-1β) and non-specific (ROS, UV radiation) signals. NF-κB is responsible for the transcription of a variety of genes that regulate inflammatory responses. The activated NF-κB complex can bind to DNA and activate the transcription of various target genes [51], many of which are inflammatory and immunoregulatory.

### 4.3. Relationship between RBP4 and Inflammatory Markers

Consistent with many previous results [52,53], significantly positive associations between RBP4 and inflammatory markers (IL-1β, CRP, TNF-α, and MCP1) were found in this study. RBP4 may induce the production proinflammatory cytokines (e.g., TNF-α, IL-6, IL-1β, MCP1) through the c-Jun N-terminal kinase pathway and the Toll-like receptor 4-mediated MPK pathway in macrophages [21], as well as in endothelial cell cytokines (VCAM, MCP1) via the NAD(P)H and NF-κB pathways [23]. On the other hand, inflammatory cytokines (e.g., IL-8) can promote RBP4 mRNA expression in adipocytes [54]; however, Kotnik et al. reported that IL-1β could decrease RBP4 production [55], although no significant associations between IL-6 and RBP4 were detected [39], and given the conflicting results, the associations of inflammatory markers and RBP4 in humans should be further examined.

There are several strengths to this study. To our knowledge, this is the first study that reported an inverse association between serum concentrations of five common carotenoids and RBP4 in a relatively large population. Our findings provided clues for the prevention of insulin resistance and inflammation-related chronic diseases via the use of carotenoid rich-foods in the diet aimed at lowering serum RBP4 levels. Next, we explored the associations of five types of carotenoids, RBP4 and five inflammatory markers and provided systematic evaluations, avoided false negative results, and found similar results among different carotenoids to reduce the probability of false positive results. 

Our study has several potential limitations. First, we could not examine a cause-and-effect relationship due to the observational study design. Second, the ELISA method used to determine RBP4 in our study measured the total RBP4 concentration and could not differentiate between holo-RBP4 (bound to retinol) and apo-RBP4 (not bound to retinol); in addition, we were unable to distinguish between the full-length and the truncated forms of RBP4 [56]. Third, although we determined the association between RBP4 and hsCRP using methods with higher precision (between-run CVs: 3.59% for RBP4 and 2.07% for hsCRP), only about a half of the total sample was screened for the other four inflammatory markers using the Human FlowCytomix methods [36] with relatively higher between-plate CVs (14.1% for MCP1, 6.6% for TNF-α, 2.5% for IL-6, and 10.2% for IL-1β). The lower precision and small sample size may also contribute to the less significant associations observed in this study. Fourth, although many covariates were controlled in our study, the concentrations of carotenoids were highest in the serum and tissue, and the serum half-life was longer than that of polyphenols; the effect of residual potential confounding factors, such as astaxanthin, vitamin D, medications which could modulate inflammation could not be ruled out.

## 5. Conclusions

In conclusion, our findings show that serum carotenoid concentrations are inversely associated with RBP4 and other inflammatory markers (hsCRP, MCP1, and TNF-α) in middle-aged and elderly Chinese men and women. Considering the pitfalls of cross-sectional studies, further experimental studies are needed to clarify the effects of carotenoids on levels of serum RBP4 and other inflammatory markers.

## Figures and Tables

**Table 1:** General and biochemical characteristics of the participants (*n* = 3031).

	*n*	Total (*n* = 3031)	Female (*n* = 2063)	Male (*n* = 968)	*p* Value
Age (years)	3031	58.6 ± 6.24	57.6 ± 5.8	60.7 ± 6.7	<0.001
BMI (kg/m^2^)	3031	23.3 ± 3.06	23.1 ± 3.11	23.8 ± 2.90	<0.001
Waist circumference (cm)	3031	83.3 ± 8.95	81.9 ± 8.90	86.2 ± 8.35	<0.001
Education (*n* (%))					0.211
Secondary school or below	869	869 (28.7)	606 (29.4)	263 (27.2)	
High school or above	2162	2162 (71.3)	1457 (70.6)	705 (72.8)	
Married (*n* (%))	2730	2730 (90.1)	1790 (86.8)	940 (97.1)	<0.001
Household income (Yuan/month/person), (*n* (%))					<0.001
≤500	57	57 (1.9)	31 (1.5)	26 (2.7)	
501–1500	790	790 (26.1)	572 (27.7)	218 (22.5)	
1501–3000	1327	1327 (43.8)	922 (44.7)	405 (41.8)	
>3000	857	857 (28.3)	538 (26.1)	319 (33.0)	
Smokers (*n* (%)) ^a^	489	489 (16.1)	6 (0.3)	483 (49.9)	<0.001
Alcohol drinkers (*n* (%)) ^b^	215	215 (7.1)	50 (2.4)	165 (17.1)	<0.001
Vitamin supplement users (*n* (%))	662	662 (21.8)	520 (25.2)	142 (14.7)	<0.001
Calcium supplement users (*n* (%))	951	951 (31.4)	757 (36.7)	194 (20.0)	<0.001
Physical activity (MET·h/day) ^c^	3031	25.8 ± 6.67	26.1 ± 6.48	25.3 ± 7.09	0.001
Serum fasting glucose (mmol/L)	2892	5.02 ± 1.16	4.97 ± 1.13	5.14 ± 1.22	<0.001
Serum fasting insulin (mU/L)	2901	9.06 ± 5.57	9.29 ± 5.73	8.58 ± 5.19	0.001
Serum TC (mmol/L)	3024	5.52 ± 1.10	5.66 ± 1.11	5.24 ± 1.03	<0.001
Serum retinol (μmol/L)	3031	1.58 (1.35, 1.86)	1.54 (1.32, 1.80)	1.68 (1.42, 1.99)	<0.001
Serum carotenoids (μmol/L)					
α-Carotene	3031	0.06 (0.04, 0.09)	0.06 (0.04, 0.10)	0.05 (0.03, 0.08)	<0.001
β-Carotene	3031	0.44 (0.25, 0.70)	0.50 (0.30, 0.77)	0.33 (0.19, 0.52)	<0.001
β-Cryptoxanthin	3031	0.12 (0.07, 0.20)	0.13 (0.08, 0.23)	0.09 (0.06, 0.16)	<0.001
Lycopene	3031	0.16 (0.10, 0.23)	0.17 (0.12, 0.25)	0.13 (0.08, 0.20)	<0.001
Lutein + zeaxanthin	3031	0.62 (0.44, 0.86)	0.64 (0.45, 0.89)	0.58 (0.41, 0.79)	<0.001
Total carotenoids	3031	1.63 (1.04, 2.08)	1.64 (1.13, 2.20)	1.28 (0.90, 1.74)	<0.001
Inflammatory marker					
MCP1 (pg/mL)	1314	454 (334, 609)	460 (339, 623)	442 (326, 575)	0.016
Interleukin-6 (pg/mL)	1312	1.31 (0.77, 2.44)	1.21 (0.73, 2.28)	1.48 (0.87, 2.63)	0.001
Interleukin-1β (pg/mL)	1313	3.88 (1.50, 17.87)	3.51 (1.40, 13.79)	6.05 (1.76, 24.20)	<0.001
TNF-α (pg/mL)	1313	6.84 (1.66, 62.43)	5.69 (1.60, 48.87)	11.44 (1.72, 71.35)	0.001
hsCRP (mg/L)	2925	0.95 (0.56, 1.93)	0.98 (0.58, 1.99)	0.86 (0.52, 1.77)	0.001
RBP4 (μg/mL)	2738	36.48 ± 6.92	35.36 ± 6.49	38.91 ± 7.19	<0.001

BMI, body mass index; MET, metabolic equivalent; TC, total cholesterol; TG, Triglycerides; MCP1, monocyte chemoattractant protein-1; TNF-α, tumor necrosis factor α; hsCRP, high sensitivity C-reactive protein; RBP4, retinol binding protein 4; Continuous variables were described by means ± standard deviation (normal distribution data) or median (Q25, Q75) (non-normal distribution data). ^a^ Smokers was defined as participants who have smoked ≥1 cigarette daily for at least six consecutive months. ^b^ Alcohol drinkers were defined as participants who have had wine ≥1 time(s) daily for at least six consecutive months. ^c^ Physical activities included daily occupational, leisure-time, and household-chores, but excluding sleeping and sitting, evaluated by metabolic equivalent (MET) hours per day.

**Table 2:** Partial correlation coefficients between serum carotenoids and inflammatory biomarkers among total participants.

	aRBP4 ^a^		RBP4		hsCRP		MCP1		IL-6		IL-1β		TNF-α	
	*r*	*p*	*r*	*p*	*r*	*p*	*r*	*p*	*r*	*p*	*r*	*p*	*r*	*p*
*n*	2734		2734		2921		1310		1292		1292		1297	
Age, sex adjusted ^M1^														
Total carotenoids ^b^	−0.129	<0.001	−0.097	<0.001	−0.159	<0.001	−0.079	0.004	0.002	0.933	0.002	0.944	−0.014	0.608
Log total carotenoids	−0.134	<0.001	−0.098	<0.001	−0.140	<0.001	−0.071	0.010	0.022	0.438	0.026	0.345	0.006	0.835
α-Carotene	−0.073	<0.001	−0.062	0.001	−0.161	<0.001	−0.088	0.001	−0.044	0.117	−0.059	0.033	−0.080	0.004
β-Carotene	−0.103	<0.001	−0.087	<0.001	−0.161	<0.001	−0.075	0.006	0.015	0.582	0.016	0.553	0.006	0.838
β-Cryptoxanthin	−0.080	<0.001	−0.057	0.003	−0.060	0.001	−0.028	0.305	0.043	0.120	0.025	0.369	0.051	0.064
Lycopene	−0.086	<0.001	−0.063	0.001	−0.136	<0.001	−0.046	0.099	−0.014	0.610	0.003	0.926	−0.033	0.231
Lutein + zeaxanthin	−0.140	<0.001	−0.093	<0.001	−0.076	<0.001	−0.059	0.033	0.009	0.758	0.022	0.424	0.004	0.890
Multivariate-adjusted ^M2^														
Total carotenoids ^b^	−0.098	<0.001	−0.079	<0.001	−0.114	<0.001	−0.090	0.001	−0.014	0.616	−0.055	0.050	−0.079	0.005
Log total carotenoids	−0.103	<0.001	−0.080	<0.001	−0.097	<0.001	−0.081	0.004	0.007	0.793	−0.033	0.242	−0.062	0.027
α-Carotene	−0.044	0.023	−0.037	0.052	−0.112	<0.001	−0.090	0.001	−0.046	0.096	−0.072	0.010	−0.096	0.001
β-Carotene	−0.067	<0.001	−0.061	0.001	−0.105	<0.001	−0.081	0.004	0.009	0.744	−0.020	0.474	−0.034	0.222
β-Cryptoxanthin	−0.067	<0.001	−0.056	0.004	−0.048	0.009	−0.036	0.200	0.026	0.350	−0.025	0.368	−0.003	0.923
Lycopene	−0.062	0.001	−0.050	0.009	−0.100	<0.001	−0.054	0.050	−0.033	0.233	−0.044	0.115	−0.089	0.001
Lutein + zeaxanthin	−0.120	<0.001	−0.084	<0.001	−0.051	0.005	−0.067	0.016	−0.006	0.834	−0.039	0.163	−0.066	0.018

^a^ retinol-adjusted RBP4 using the residual method. ^b^ All variables were the Z-score of log transformed value, but only Z-score for total carotenoids. ^M1^ adjusted for age and gender; ^M2^ adjusted for age, gender, marital status, education level, household income (Yuan/month per person), smoking, alcohol drinking, vitamin supplements, MET (exclude sleeping and rest), TC, and BMI.

**Table 3:** Partial correlation coefficients between serum carotenoids and inflammatory biomarkers by gender.

	Age, Sex Adjusted ^M1^							Multivariate-Adjusted ^M2^						
	aRBP4 ^d^	RBP4	hsCRP	MCP1	IL6	IL-1β	TNF-α	aRBP4 ^d^	RBP4	hsCRP	MCP1	IL-6	IL-1β	TNF-α
**Women**														
***n***	1866	1866	1989	894	879	880	883	1866	1866	1989	894	879	880	883
Total carotenoids ^e^	−0.143 ^c^	−0.112 ^c^	−0.155 ^c^	−0.043	0.035	−0.002	0.001	−0.111 ^c^	−0.091 ^c^	−0.110 ^c^	−0.053	0.029	−0.059	−0.061
Log total carotenoids	−0.146 ^c^	−0.111 ^c^	−0.146 ^c^	−0.045	0.057	0.037	0.027	−0.115 ^c^	−0.093 ^c^	−0.101 ^c^	−0.054	0.052	−0.022	−0.036
α-Carotene	−0.071 ^b^	−0.058 ^a^	−0.140 ^c^	−0.068 ^a^	−0.027	−0.074 ^a^	−0.075 ^a^	−0.039	−0.03	−0.088	−0.069 ^a^	−0.025	−0.091 ^b^	−0.094 ^b^
β-Carotene	−0.116 ^c^	−0.095 ^c^	−0.171 ^c^	−0.064	0.039	0.039	0.028	−0.079 ^c^	−0.068 ^b^	−0.113 ^c^	−0.068 ^a^	0.041	0.003	−0.008
β-Cryptoxanthin	−0.090 ^c^	−0.073 ^b^	−0.055 ^a^	0.014	0.091 ^b^	0.021	0.055	−0.078 ^c^	−0.070 ^b^	−0.052 ^a^	0.001	0.079 ^a^	−0.025	0.002
Lycopene	−0.109 ^c^	−0.088 ^c^	−0.127 ^c^	−0.002	−0.009	−0.023	−0.017	−0.082 ^c^	−0.071 ^b^	−0.089 ^c^	−0.01	−0.020	−0.069 ^a^	−0.068 ^a^
Lutein + zeaxanthin	−0.149 ^c^	−0.106 ^c^	−0.086 ^c^	−0.042	0.035	0.028	0.012	−0.129 ^c^	−0.097 ^c^	−0.059 ^b^	−0.05	0.029	−0.035	−0.056
**Men**														
***n***	866	866	930	414	411	410	412	866	866	930	414	411	410	412
Total carotenoids ^e^	−0.096 ^b^	−0.059	−0.165 ^c^	−0.148 ^b^	−0.06	0.013	−0.041	−0.066	−0.043	−0.124 ^c^	−0.160 ^c^	−0.096	−0.042	−0.109 ^a^
Log total carotenoids	−0.105 ^b^	−0.063	−0.124 ^c^	−0.121 ^a^	−0.046	0.007	−0.035	−0.075 ^a^	−0.047	−0.089 ^b^	−0.131 ^b^	−0.082	−0.049	−0.105 ^a^
α-Carotene	−0.076 ^a^	−0.068 ^a^	−0.206 ^c^	−0.126 ^b^	−0.074	−0.029	−0.088	−0.051	−0.048	−0.160 ^c^	−0.131 ^b^	−0.082	−0.033	−0.095
β-Carotene	−0.072 ^a^	−0.062	−0.134 ^c^	−0.091	−0.027	−0.025	−0.034	−0.036	−0.038	−0.090 ^b^	−0.099 ^a^	−0.05	−0.061	−0.076
β-Cryptoxanthin	−0.055	−0.019	−0.067 ^a^	−0.119 ^a^	−0.061	0.038	0.048	−0.04	−0.017	−0.046	−0.123 ^a^	−0.094	−0.023	−0.016
Lycopene	−0.032	−0.005	−0.154 ^c^	−0.128 ^b^	−0.021	0.056	−0.062	−0.012	0.003	−0.117 ^c^	−0.136 ^b^	−0.049	0.007	−0.127 ^b^
Lutein + zeaxanthin	−0.121 ^c^	−0.065	−0.055	−0.093	−0.045	0.011	−0.011	−0.100 ^b^	−0.055	−0.038	−0.098 ^a^	−0.077	−0.04	−0.077

^a^ *p* < 0.05; ^b^ *p* < 0.01; ^c^ *p* < 0.001. ^d^ retinol-adjusted RBP4 using the residual method. ^e^ All variables were the Z-score of log transformed value, but only Z-score for total carotenoids. ^M1^ adjusted for age and gender. ^M2^ adjusted for age, gender, marital status, education level, household income (Yuan/month per person), smoking, alcohol drinking, vitamin supplements, MET (exclude sleeping and rest), TC, and BMI.

## Appendix

**Table A1:** Partial correlation coefficients between serum carotenoids and inflammatory biomarkers by gender after further adjusted for some medications.

	Age, Sex Adjusted ^M1^							Multivariate-Adjusted ^M2^						
	aRBP4 ^d^	RBP4	hsCRP	MCP1	IL6	IL-1β	TNF-α	aRBP4 ^d^	RBP4	hsCRP	MCP1	IL-6	IL-1β	TNF-α
Women	1866	1866	1989	894	879	880	883	1866	1866	1989	894	879	880	883
Total carotenoids ^e^	−0.143 ^c^	−0.112 ^c^	−0.155 ^c^	−0.043	0.035	−0.002	0.001	−0.111 ^c^	−0.091 ^c^	−0.107 ^c^	−0.048	0.032	−0.036	−0.037
Log total carotenoids	−0.146 ^c^	−0.111 ^c^	−0.146 ^c^	−0.045	0.057	0.037	0.027	−0.115 ^c^	−0.093 ^c^	−0.097 ^c^	−0.052	0.051	−0.010	−0.026
α-Carotene	−0.071 ^b^	−0.058 ^a^	−0.140 ^c^	−0.068 ^a^	−0.027	−0.074 ^a^	−0.075 ^a^	−0.039	−0.030	−0.086 ^c^	−0.054	−0.009	−0.055	−0.053
β-Carotene	−0.116 ^c^	−0.095 ^c^	−0.171 ^c^	−0.064	0.039	0.039	0.028	−0.079 ^c^	−0.068 ^b^	−0.110 ^c^	−0.066	0.040	0.016	−0.004
β-Cryptoxanthin	−0.090 ^c^	−0.073 ^b^	−0.055 ^a^	0.014	0.091 ^b^	0.021	0.055	−0.077 ^c^	−0.069 ^b^	−0.051 ^a^	−0.005	0.072 ^a^	−0.017	0.009
Lycopene	−0.109 ^c^	−0.088 ^c^	−0.127 ^c^	−0.002	−0.009	−0.023	−0.017	−0.082 ^c^	−0.069 ^b^	−0.086 ^c^	−0.0004	−0.011	−0.033	−0.028
Lutein + zeaxanthin	−0.149 ^c^	−0.106 ^c^	−0.086 ^c^	−0.042	0.035	0.028	0.012	−0.130 ^c^	−0.098 ^c^	−0.056 ^b^	−0.050	0.025	−0.041	−0.065
Men	866	866	930	414	411	410	412	866	866	930	414	411	410	412
Total carotenoids ^e^	−0.096 ^b^	−0.059	−0.165 ^c^	−0.148 ^b^	−0.06	0.013	−0.041	−0.068	−0.043	−0.123 ^c^	−0.156 ^b^	−0.087	−0.029	−0.099 ^a^
Log total carotenoids	−0.105 ^b^	−0.063	−0.124 ^c^	−0.121 ^a^	−0.046	0.007	−0.035	−0.078 ^a^	−0.047	−0.088 ^b^	−0.127 ^b^	−0.072	−0.042	−0.100 ^a^
α-Carotene	−0.076 ^a^	−0.068 ^a^	−0.206 ^c^	−0.126 ^b^	−0.074	−0.029	−0.088	−0.052	−0.049	−0.160 ^c^	−0.125 ^b^	−0.076	−0.015	−0.079
β-Carotene	−0.072 ^a^	−0.062	−0.134 ^c^	−0.091	−0.027	−0.025	−0.034	−0.038	−0.037	−0.090 ^b^	−0.096	−0.051	−0.074	−0.089
β-Cryptoxanthin	−0.055	−0.019	−0.067 ^a^	−0.119 ^a^	−0.061	0.038	0.048	−0.040	−0.016	−0.044	−0.126 ^a^	−0.095	−0.024	−0.017
Lycopene	−0.032	−0.005	−0.154 ^c^	−0.128 ^b^	−0.021	0.056	−0.062	−0.013	0.003	−0.117 ^c^	−0.125 ^b^	−0.028	0.041	−0.096
Lutein + zeaxanthin	−0.121 ^c^	−0.065	−0.055	−0.093	−0.045	0.011	−0.011	−0.104 ^b^	−0.057	−0.036	−0.098 ^a^	−0.070	−0.036	−0.074

^a^ *p* < 0.05; ^b^ *p* < 0.01; ^c^ *p* < 0.001. ^d^ retinol-adjusted RBP4 using the residual method. ^e^ All variables were the *Z*-score of log transformed value, but only *Z*-score for total carotenoids. ^M1^ adjusted for age and gender; ^M2^ adjusted for age, gender, marital status, education level, household income (Yuan/month per person), smoking, drinking, vitamin supplements, MET (exclude sleeping and rest), TC, BMI, and using of drugs (e.g., statin, anti-hypertension or anti-diabetic drugs).

**Table A2:** Unadjusted and adjusted associations between serum carotenoids and the presence of high inflammation determined by hsCRP and RBP4 ^a^.

Carotenoids (μmol/L) ^b^	hsCRP (mg/L)				RBP4 (μg/mL)			
	Model 1		Model 2		Model 1		Model 2	
	OR (95% CI)	*p*	OR (95% CI)	*p*	OR (95% CI)	*p*	OR (95% CI)	*p*
Total carotenoids	0.31 (0.22–0.45)	<0.001	0.42 (0.29–0.61)	<0.001	0.42 (0.27–0.64)	<0.001	0.43 (0.27–0.67)	<0.001
α-Carotene	0.38 (0.29–0.49)	<0.001	0.48 (0.37–0.63)	<0.001	0.60 (0.43–0.82)	<0.001	0.64 (0.46–0.90)	0.009
β-Carotene	0.39 (0.30–0.49)	<0.001	0.51 (0.40–0.67)	<0.001	0.64 (0.48–0.85)	<0.001	0.68 (0.50–0.93)	0.015
β-Cryptoxanthin	0.72 (0.57–0.91)	0.006	0.76 (0.59–0.97)	0.029	0.70 (0.52–0.95)	<0.001	0.75 (0.55–1.01)	0.060
Lycopene	0.38 (0.28–0.50)	<0.001	0.46 (0.34–0.62)	<0.001	0.54 (0.38–0.76)	<0.001	0.54 (0.38–0.77)	0.001
Lutein + zeaxanthin	0.60 (0.43–0.83)	0.002	0.73 (0.52–1.04)	0.085	0.44 (0.29–0.65)	<0.001	0.44 (0.29–0.68)	<0.001

OR: odds ratio; CI: confidence interval; Ref.: referent group; TC: total cholesterol. ^a^ Elevated inflammation levels which determined by hsCRP (≥1.0 mg/L) [38] and RBP4 (arbitrarily defined as top quintile by sex) (40.62 μg/mL in female and 44.70 μg/mL in male). ^b^ All variables were the log transformed values. (Model 1): adjusted for age and gender; (Model 2), adjusted for age, gender, marital status, education level, household income (Yuan/month per person), smoking, drinking, vitamin supplements, MET (exclude sleeping and rest), TC, and BMI.
